# Bis{μ-2,2′-[*o*-phenyl­enebis(nitrilo­methyl­idyne)]diphenolato}dicopper(II) *N*,*N*′-dimethyl­formamide disolvate

**DOI:** 10.1107/S1600536808005394

**Published:** 2008-02-29

**Authors:** Guofeng Yu, Yu Ding, Li Wang, Zhengbing Fu, Xinliang Hu

**Affiliations:** a Department of Chemistry, Xiaogan University, Xiaogan, Hubei 432000, People’s Republic of China

## Abstract

The title compound, [Cu_2_(C_20_H_14_N_2_O_2_)_2_]·2C_3_H_7_NO, consists of a centrosymmetric dimer composed of two copper(II) ions and two tetra­dentate salphen ligands {H_2_salphen is 2,2′-[*o*-phenyl­enebis(nitrilo­methyl­idyne)]diphenol}, and two dimethyl­formamide solvent mol­ecules. The Cu^II^ atom is bonded to two N imino atoms and three phenolate O atoms of salphen. One deprotonated phenol group of each ligand bridges two Cu atoms, forming the dimer. The geometry about the five-coordinate Cu atom can best be described as slightly distorted recta­ngular pyramidal. The crystal structure is stabilized by π–π inter­actions [centroid-centroid distance 3.779 (2) Å] and C—H⋯O hydrogen bonds.

## Related literature

For related literature, see: Suzuki *et al.* (1997 ▶).
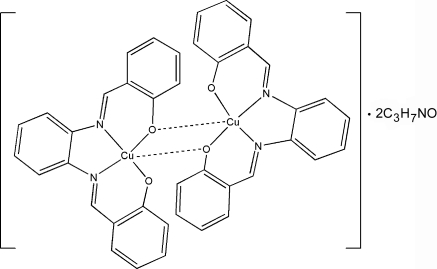


## Experimental

### 

#### Crystal data

[Cu_2_(C_20_H_14_N_2_O_2_)_2_]·2C_3_H_7_NO
*M* *_r_* = 901.94
Monoclinic, 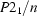

*a* = 8.1864 (5) Å
*b* = 14.792 (1) Å
*c* = 16.9584 (11) Å
β = 93.252 (1)°
*V* = 2050.2 (2) Å^3^
*Z* = 2
Mo *K*α radiation
μ = 1.09 mm^−1^
*T* = 294 (2) K
0.20 × 0.10 × 0.10 mm

#### Data collection

Bruker SMART CCD area-detector diffractometer
Absorption correction: multi-scan (*SADABS*; Sheldrick, 2001 ▶) *T*_min_ = 0.811, *T*_max_ = 0.898
13976 measured reflections
4468 independent reflections
3126 reflections with *I* > 2σ(*I*)
*R*_int_ = 0.083

#### Refinement

*R*[*F* ^2^ > 2σ(*F* ^2^)] = 0.051
*wR*(*F* ^2^) = 0.129
*S* = 0.98
4468 reflections
273 parameters
H-atom parameters constrained
Δρ_max_ = 0.52 e Å^−3^
Δρ_min_ = −0.36 e Å^−3^

### 

Data collection: *SMART* (Bruker, 2001 ▶); cell refinement: *SAINT-Plus* (Bruker, 2001 ▶); data reduction: *SAINT-Plus*; program(s) used to solve structure: *SHELXS97* (Sheldrick, 2008 ▶); program(s) used to refine structure: *SHELXL97* (Sheldrick, 2008 ▶); molecular graphics: *SHELXTL* (Sheldrick, 2008 ▶); software used to prepare material for publication: *SHELXTL*.

## Supplementary Material

Crystal structure: contains datablocks I, global, New_Global_Publ_Block_1. DOI: 10.1107/S1600536808005394/br2066sup1.cif

Structure factors: contains datablocks I. DOI: 10.1107/S1600536808005394/br2066Isup2.hkl

Additional supplementary materials:  crystallographic information; 3D view; checkCIF report

## Figures and Tables

**Table 1:** Selected bond lengths (Å)

Cu1—O1	1.907 (2)
Cu1—O2	1.909 (2)
Cu1—N1	1.946 (2)
Cu1—N2	1.950 (2)
Cu1—O1^i^	2.783 (11).

Symmetry code: (i) 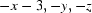.

**Table 2:** Hydrogen-bond geometry (Å, °)

*D*—H⋯*A*	*D*—H	H⋯*A*	*D*⋯*A*	*D*—H⋯*A*
C14—H14⋯O3	0.93	2.44	3.244 (4)	145
C5—H5⋯O3	0.93	2.54	3.333 (4)	144
C23—H23⋯O2^ii^	0.93	2.58	3.457 (5)	158
C7—H7⋯O3^iii^	0.93	2.41	3.333 (4)	170
C2—H2⋯O3^iii^	0.93	2.47	3.389 (4)	172
C21—H21*A*⋯O3	0.96	2.36	2.756 (5)	104

Symmetry codes: (ii) 
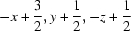
; (iii) 
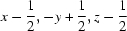
.

**Fig. 1.**
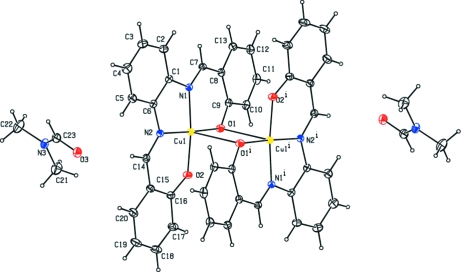
The molecular structure of (I), showing ellipsoids at the 50% probability level.

**Fig. 2.**
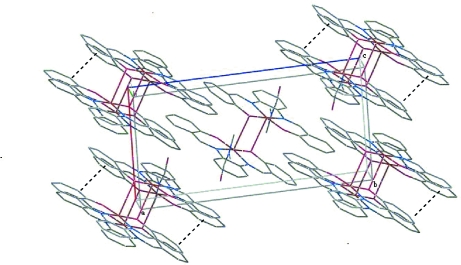
The molecular packing diagram of (I).

## supplementary crystallographic information

### Comment

The stucture of the title compound, [Cu(salphen)]_2_˙2DMF, (DMF= *N*,*N*'-dimethylformamide), (I), is shown in Fig.1.

The salphen derivatives and their manganese complexes have been synthesized and characterized(Suzuki *et al.*,1997).

Herein, we report the crystal structure of such a compound. As shown in Fig.1, the molecular structure of the title compound is constructed of a centrosymmetric dimer in which the copper(II) atoms are linked by µ-phenoxo bridges from one of the phenolic oxygen atoms of each salphen ligand to the opposite metal center. The distance of Cu1···Cu1(2 - *x*,-*y*,-*z*) separation and the angles of Cu1–O1–Cu1(2 - *x*,-*y*,-*z*)are 3.436 Å, and 92.19°, respectively. Two nitrogen atoms and two oxygen atoms from salphen ligands occupy the coordination sites about each copper. The apical Cu–O (phenoxo) and Cu–N (imine) (see Table 2) bond distances are somewhat shorter than the long equatorial Cu1—O1 distance. The basal atoms about the two copper atoms are coplanar; consequently, the environment around each copper atom can be described as a distorted triangular pyramid.

The centroid-centroid distance between the C8—C13 benzene ring (centroid *Cg*1) belonging to one salicylaldehyde ring system in one dimer and the C15—C20 benzene ring (centroid *Cg*2) of the salicylaldehyde ring system from the neighboring dimer at (2 - *x*, -*y*, -*z*) is 3.779 (2), and the dihedral angles (between planes *Cg*1 and *Cg*2) and (between planes *Cg*1- *Cg*2 vector and the normal to the C8—C13 ring) are 10.82 and 16.52°, respectively; these values indicate the existence of significant π-π stacking interactions between adjacent rings, as shown in Fig.2, which stabilizes the crystal structure together with the hydrogen bonds.

### Experimental

[Cu(C_20_H_14_N_2_O_2_)]_2 _.2DMF was prepared as followings: to a solution of H_2_salphen 0.158 mg(0.5 mmol) in methanol (20 mL) and DMF(20 mL) was added Cu(OAc)_2_˙2H_2_O(0.113 g, 0.5 mmol). After the mixture was stirred for half an hour, the solution was filtered. The filtrate was kept for several days at ambient temperature, and green-black block crystals were obtained.

### Refinement

The H atoms bonded to C atoms were introduced at calculated positions and refined using a riding model, with *U*_iso_(H) = 1.2*U*_eq_(C) and *U*_iso_(H) = 1.5*U*_eq_(C), and C–H distances of 0.93–0.96 Å.

### Crystal data

**Table:**

[Cu_2_(C_20_H_14_N_2_O_2_)_2_]·2C_3_H_7_NO	*F*_000_ = 932
*M**_r_* = 901.94	*D*_x_ = 1.461 Mg m^−^^3^
Monoclinic, *P*2_1_/*n*	Mo *K*α radiation λ = 0.71073 Å
Hall symbol: -P 2yn	Cell parameters from 3178 reflections
*a* = 8.1864 (5) Å	θ = 2.8–23.0º
*b* = 14.7920 (10) Å	µ = 1.10 mm^−^^1^
*c* = 16.9584 (11) Å	*T* = 294 (2) K
β = 93.252 (1)º	Block, black
*V* = 2050.2 (2) Å^3^	0.20 × 0.10 × 0.10 mm
*Z* = 2	

### Data collection

**Table:**

Bruker SMART CCD area-detector diffractometer	4468 independent reflections
Radiation source: fine-focus sealed tube	3126 reflections with *I* > 2σ(*I*)
Monochromator: graphite	*R*_int_ = 0.083
*T* = 294(2) K	θ_max_ = 27.0º
φ and ω scans	θ_min_ = 1.8º
Absorption correction: multi-scan(SADABS; Sheldrick, 2001)	*h* = −10→10
*T*_min_ = 0.811, *T*_max_ = 0.898	*k* = −14→18
13976 measured reflections	*l* = −20→21

### Refinement

**Table:**

Refinement on *F*^2^	Secondary atom site location: difference Fourier map
Least-squares matrix: full	Hydrogen site location: inferred from neighbouring sites
*R*[*F*^2^ > 2σ(*F*^2^)] = 0.051	H-atom parameters constrained
*wR*(*F*^2^) = 0.129	*w* = 1/[σ^2^(*F*_o_^2^) + (0.0609*P*)^2^] where *P* = (*F*_o_^2^ + 2*F*_c_^2^)/3
*S* = 0.98	(Δ/σ)_max_ < 0.001
4468 reflections	Δρ_max_ = 0.52 e Å^−^^3^
273 parameters	Δρ_min_ = −0.36 e Å^−^^3^
Primary atom site location: structure-invariant direct methods	Extinction correction: none

### Special details

**Table:**

Geometry. All e.s.d.'s (except the e.s.d. in the dihedral angle between two l.s. planes) are estimated using the full covariance matrix. The cell e.s.d.'s are taken into account individually in the estimation of e.s.d.'s in distances, angles and torsion angles; correlations between e.s.d.'s in cell parameters are only used when they are defined by crystal symmetry. An approximate (isotropic) treatment of cell e.s.d.'s is used for estimating e.s.d.'s involving l.s. planes.
Refinement. Refinement of *F*^2^ against ALL reflections. The weighted *R*-factor *wR* and goodness of fit *S* are based on *F*^2^, conventional *R*-factors *R* are based on *F*, with *F* set to zero for negative *F*^2^. The threshold expression of *F*^2^ > σ(*F*^2^) is used only for calculating *R*-factors(gt) *etc*. and is not relevant to the choice of reflections for refinement. *R*-factors based on *F*^2^ are statistically about twice as large as those based on *F*, and *R*- factors based on ALL data will be even larger.

### Fractional atomic coordinates and isotropic or equivalent isotropic displacement parameters (Å^2^)

**Table:**

	*x*	*y*	*z*	*U*_iso_*/*U*_eq_	
Cu1	0.87999 (4)	0.02801 (2)	0.07623 (2)	0.03962 (15)	
C1	0.7432 (3)	0.2022 (2)	0.05569 (18)	0.0394 (7)	
C2	0.6560 (4)	0.2775 (2)	0.0271 (2)	0.0506 (8)	
H2	0.5941	0.2742	−0.0205	0.061*	
C3	0.6622 (5)	0.3569 (2)	0.0699 (2)	0.0614 (10)	
H3	0.6053	0.4075	0.0508	0.074*	
C4	0.7528 (5)	0.3612 (2)	0.1410 (3)	0.0688 (11)	
H4	0.7547	0.4146	0.1700	0.083*	
C5	0.8397 (4)	0.2880 (2)	0.1695 (2)	0.0546 (9)	
H5	0.9014	0.2921	0.2171	0.065*	
C6	0.8359 (3)	0.2075 (2)	0.12719 (18)	0.0392 (7)	
C7	0.6479 (4)	0.0977 (2)	−0.04292 (18)	0.0399 (7)	
H7	0.5858	0.1452	−0.0647	0.048*	
C8	0.6304 (4)	0.0124 (2)	−0.08095 (17)	0.0412 (7)	
C9	0.7215 (4)	−0.0655 (2)	−0.05630 (19)	0.0419 (7)	
C10	0.6853 (4)	−0.1464 (2)	−0.0970 (2)	0.0509 (8)	
H10	0.7399	−0.1991	−0.0812	0.061*	
C11	0.5710 (4)	−0.1499 (3)	−0.1597 (2)	0.0585 (10)	
H11	0.5500	−0.2046	−0.1853	0.070*	
C12	0.4865 (4)	−0.0729 (3)	−0.1852 (2)	0.0566 (9)	
H12	0.4112	−0.0753	−0.2283	0.068*	
C13	0.5161 (4)	0.0058 (2)	−0.1460 (2)	0.0495 (9)	
H13	0.4588	0.0573	−0.1627	0.059*	
C14	0.9997 (4)	0.1198 (2)	0.21836 (19)	0.0473 (8)	
H14	1.0089	0.1718	0.2492	0.057*	
C15	1.0785 (4)	0.0411 (2)	0.2491 (2)	0.0478 (8)	
C16	1.0733 (4)	−0.0430 (2)	0.2097 (2)	0.0483 (9)	
C17	1.1600 (5)	−0.1157 (3)	0.2469 (2)	0.0654 (11)	
H17	1.1591	−0.1720	0.2226	0.078*	
C18	1.2455 (5)	−0.1043 (3)	0.3184 (3)	0.0746 (12)	
H18	1.3026	−0.1529	0.3411	0.090*	
C19	1.2486 (5)	−0.0225 (3)	0.3570 (2)	0.0787 (14)	
H19	1.3061	−0.0159	0.4055	0.094*	
C20	1.1659 (5)	0.0486 (3)	0.3230 (2)	0.0652 (11)	
H20	1.1671	0.1038	0.3493	0.078*	
C21	0.7335 (6)	0.1052 (3)	0.4145 (3)	0.0950 (15)	
H21A	0.8299	0.0998	0.3852	0.142*	
H21B	0.6512	0.0643	0.3932	0.142*	
H21C	0.7597	0.0905	0.4689	0.142*	
C22	0.5134 (5)	0.2143 (3)	0.4363 (3)	0.0993 (17)	
H22A	0.4918	0.2780	0.4326	0.149*	
H22B	0.5093	0.1953	0.4902	0.149*	
H22C	0.4325	0.1820	0.4042	0.149*	
C23	0.7679 (5)	0.2604 (3)	0.3829 (2)	0.0601 (10)	
H23	0.7243	0.3184	0.3800	0.072*	
N1	0.7417 (3)	0.11587 (16)	0.01899 (14)	0.0367 (6)	
N2	0.9162 (3)	0.12659 (17)	0.15158 (14)	0.0384 (6)	
N3	0.6731 (4)	0.1958 (2)	0.40887 (18)	0.0592 (8)	
O1	0.8363 (3)	−0.06591 (14)	0.00094 (13)	0.0492 (6)	
O2	0.9963 (3)	−0.05820 (15)	0.14172 (14)	0.0524 (6)	
O3	0.9070 (3)	0.25103 (17)	0.36246 (15)	0.0639 (7)	

### Atomic displacement parameters (Å^2^)

**Table:**

	*U*^11^	*U*^22^	*U*^33^	*U*^12^	*U*^13^	*U*^23^
Cu1	0.0424 (2)	0.0340 (2)	0.0420 (2)	0.00078 (17)	−0.00157 (16)	0.00026 (17)
C1	0.0368 (16)	0.0327 (17)	0.0494 (18)	−0.0021 (13)	0.0080 (13)	0.0017 (14)
C2	0.052 (2)	0.0390 (19)	0.060 (2)	−0.0029 (16)	−0.0039 (16)	0.0066 (17)
C3	0.069 (2)	0.035 (2)	0.080 (3)	0.0043 (17)	−0.001 (2)	0.0036 (19)
C4	0.094 (3)	0.033 (2)	0.080 (3)	−0.002 (2)	0.004 (2)	−0.011 (2)
C5	0.067 (2)	0.044 (2)	0.052 (2)	−0.0055 (17)	−0.0006 (17)	−0.0067 (17)
C6	0.0384 (16)	0.0331 (18)	0.0468 (18)	−0.0044 (13)	0.0090 (13)	−0.0004 (14)
C7	0.0396 (16)	0.0368 (18)	0.0431 (17)	0.0010 (14)	0.0015 (13)	0.0054 (14)
C8	0.0402 (17)	0.0428 (19)	0.0410 (18)	−0.0072 (14)	0.0065 (13)	−0.0002 (15)
C9	0.0402 (17)	0.0417 (19)	0.0445 (18)	−0.0069 (15)	0.0090 (14)	−0.0039 (15)
C10	0.054 (2)	0.043 (2)	0.057 (2)	−0.0049 (16)	0.0113 (17)	−0.0072 (17)
C11	0.059 (2)	0.060 (3)	0.058 (2)	−0.0212 (19)	0.0127 (18)	−0.0195 (19)
C12	0.048 (2)	0.072 (3)	0.049 (2)	−0.0140 (19)	0.0005 (16)	−0.012 (2)
C13	0.0421 (19)	0.057 (2)	0.049 (2)	−0.0032 (16)	0.0006 (15)	0.0002 (17)
C14	0.0434 (18)	0.054 (2)	0.0449 (19)	−0.0027 (16)	0.0038 (15)	−0.0066 (16)
C15	0.0367 (17)	0.060 (2)	0.0468 (19)	−0.0023 (15)	0.0010 (14)	0.0082 (17)
C16	0.0377 (17)	0.056 (2)	0.051 (2)	−0.0013 (15)	0.0059 (15)	0.0180 (17)
C17	0.067 (2)	0.060 (3)	0.068 (3)	0.004 (2)	0.001 (2)	0.024 (2)
C18	0.068 (3)	0.080 (3)	0.075 (3)	0.003 (2)	−0.006 (2)	0.039 (3)
C19	0.071 (3)	0.108 (4)	0.055 (2)	−0.005 (3)	−0.016 (2)	0.026 (3)
C20	0.062 (2)	0.083 (3)	0.049 (2)	−0.005 (2)	−0.0043 (18)	0.007 (2)
C21	0.113 (4)	0.057 (3)	0.119 (4)	0.005 (3)	0.038 (3)	0.011 (3)
C22	0.066 (3)	0.095 (4)	0.140 (5)	−0.001 (3)	0.032 (3)	−0.015 (3)
C23	0.073 (3)	0.047 (2)	0.060 (2)	0.001 (2)	0.002 (2)	−0.0103 (19)
N1	0.0376 (13)	0.0332 (14)	0.0393 (14)	−0.0014 (11)	0.0030 (11)	0.0017 (11)
N2	0.0356 (13)	0.0390 (15)	0.0405 (14)	−0.0020 (11)	0.0026 (11)	−0.0006 (12)
N3	0.0649 (19)	0.0447 (19)	0.070 (2)	0.0021 (15)	0.0183 (16)	−0.0075 (16)
O1	0.0563 (14)	0.0337 (12)	0.0564 (14)	0.0036 (11)	−0.0077 (11)	−0.0049 (11)
O2	0.0599 (15)	0.0405 (13)	0.0557 (14)	0.0052 (11)	−0.0063 (11)	0.0072 (11)
O3	0.0575 (16)	0.0729 (18)	0.0617 (16)	−0.0080 (14)	0.0076 (13)	−0.0121 (14)

### Geometric parameters (Å, °)

**Table:**

Cu1—O1	1.907 (2)	C12—C13	1.355 (5)
Cu1—O2	1.909 (2)	C12—H12	0.9300
Cu1—N1	1.946 (2)	C13—H13	0.9300
Cu1—N2	1.950 (2)	C14—N2	1.293 (4)
Cu1—O1^i^	2.783 (11).	C14—C15	1.416 (4)
C1—C2	1.395 (4)	C14—H14	0.9300
C1—C6	1.396 (4)	C15—C20	1.411 (5)
C1—N1	1.420 (4)	C15—C16	1.412 (5)
C2—C3	1.380 (5)	C16—O2	1.302 (4)
C2—H2	0.9300	C16—C17	1.416 (4)
C3—C4	1.381 (6)	C17—C18	1.375 (6)
C3—H3	0.9300	C17—H17	0.9300
C4—C5	1.369 (5)	C18—C19	1.375 (6)
C4—H4	0.9300	C18—H18	0.9300
C5—C6	1.389 (4)	C19—C20	1.360 (6)
C5—H5	0.9300	C19—H19	0.9300
C6—N2	1.416 (4)	C20—H20	0.9300
C7—N1	1.294 (4)	C21—N3	1.431 (5)
C7—C8	1.421 (4)	C21—H21A	0.9600
C7—H7	0.9300	C21—H21B	0.9600
C8—C13	1.409 (4)	C21—H21C	0.9600
C8—C9	1.423 (5)	C22—N3	1.438 (5)
C9—O1	1.312 (4)	C22—H22A	0.9600
C9—C10	1.405 (4)	C22—H22B	0.9600
C10—C11	1.376 (5)	C22—H22C	0.9600
C10—H10	0.9300	C23—O3	1.216 (4)
C11—C12	1.389 (5)	C23—N3	1.321 (5)
C11—H11	0.9300	C23—H23	0.9300
			
O1—Cu1—O2	88.35 (10)	N2—C14—H14	116.8
O1—Cu1—N1	94.06 (10)	C15—C14—H14	116.8
O2—Cu1—N1	173.09 (10)	C20—C15—C16	119.3 (3)
O1—Cu1—N2	177.59 (9)	C20—C15—C14	117.4 (4)
O2—Cu1—N2	93.78 (10)	C16—C15—C14	123.3 (3)
N1—Cu1—N2	83.69 (10)	O2—C16—C15	124.8 (3)
C2—C1—C6	119.9 (3)	O2—C16—C17	118.0 (3)
C2—C1—N1	125.1 (3)	C15—C16—C17	117.2 (3)
C6—C1—N1	115.0 (3)	C18—C17—C16	121.1 (4)
C3—C2—C1	119.6 (3)	C18—C17—H17	119.4
C3—C2—H2	120.2	C16—C17—H17	119.4
C1—C2—H2	120.2	C17—C18—C19	121.5 (4)
C2—C3—C4	120.0 (3)	C17—C18—H18	119.3
C2—C3—H3	120.0	C19—C18—H18	119.3
C4—C3—H3	120.0	C20—C19—C18	118.8 (4)
C5—C4—C3	120.9 (4)	C20—C19—H19	120.6
C5—C4—H4	119.5	C18—C19—H19	120.6
C3—C4—H4	119.5	C19—C20—C15	122.1 (4)
C4—C5—C6	120.0 (3)	C19—C20—H20	119.0
C4—C5—H5	120.0	C15—C20—H20	119.0
C6—C5—H5	120.0	N3—C21—H21A	109.5
C5—C6—C1	119.5 (3)	N3—C21—H21B	109.5
C5—C6—N2	125.2 (3)	H21A—C21—H21B	109.5
C1—C6—N2	115.2 (3)	N3—C21—H21C	109.5
N1—C7—C8	126.3 (3)	H21A—C21—H21C	109.5
N1—C7—H7	116.8	H21B—C21—H21C	109.5
C8—C7—H7	116.8	N3—C22—H22A	109.5
C13—C8—C7	117.5 (3)	N3—C22—H22B	109.5
C13—C8—C9	119.2 (3)	H22A—C22—H22B	109.5
C7—C8—C9	123.3 (3)	N3—C22—H22C	109.5
O1—C9—C10	118.9 (3)	H22A—C22—H22C	109.5
O1—C9—C8	124.2 (3)	H22B—C22—H22C	109.5
C10—C9—C8	116.9 (3)	O3—C23—N3	126.1 (4)
C11—C10—C9	121.8 (3)	O3—C23—H23	116.9
C11—C10—H10	119.1	N3—C23—H23	116.9
C9—C10—H10	119.1	C7—N1—C1	122.1 (3)
C10—C11—C12	121.0 (3)	C7—N1—Cu1	124.6 (2)
C10—C11—H11	119.5	C1—N1—Cu1	113.06 (19)
C12—C11—H11	119.5	C14—N2—C6	122.3 (3)
C13—C12—C11	118.6 (3)	C14—N2—Cu1	124.7 (2)
C13—C12—H12	120.7	C6—N2—Cu1	112.94 (19)
C11—C12—H12	120.7	C23—N3—C21	119.5 (3)
C12—C13—C8	122.4 (4)	C23—N3—C22	122.2 (3)
C12—C13—H13	118.8	C21—N3—C22	118.3 (3)
C8—C13—H13	118.8	C9—O1—Cu1	126.3 (2)
N2—C14—C15	126.4 (3)	C16—O2—Cu1	126.9 (2)

Symmetry codes: (i) −*x*−3, −*y*, −*z*.

### Hydrogen-bond geometry (Å, °)

**Table:**

*D*—H···*A*	*D*—H	H···*A*	*D*···*A*	*D*—H···*A*
C14—H14···O3	0.93	2.44	3.244 (4)	145
C5—H5···O3	0.93	2.54	3.333 (4)	144
C23—H23···O2^ii^	0.93	2.58	3.457 (5)	158
C7—H7···O3^iii^	0.93	2.41	3.333 (4)	170
C2—H2···O3^iii^	0.93	2.47	3.389 (4)	172
C21—H21A···O3	0.96	2.36	2.756 (5)	104

Symmetry codes: (ii) −*x*+3/2, *y*+1/2, −*z*+1/2; (iii) *x*−1/2, −*y*+1/2, *z*−1/2.
